# The Final Step in the Application of Perovskite Solar Cells

**DOI:** 10.3390/ma15072554

**Published:** 2022-03-31

**Authors:** Jiangshan Feng

**Affiliations:** Key Laboratory of Applied Surface and Colloid Chemistry, Ministry of Education, School of Materials Science and Engineering, Shaanxi Key Laboratory for Advanced Energy Devices, Shaanxi Engineering Lab for Advanced Energy Technology, Institute for Advanced Energy Materials, Shaanxi Normal University, Xi’an 710119, China; fengjs@snnu.edu.cn

Since 2009, there has been renewed interest in perovskite materials due to the rise of metal halide perovskite photoelectric materials, especially in the field of perovskite solar cells (PSCs). Rapid development has been achieved in the last ten years regarding the photoelectric conversion efficiency (PCE) of PSC; at present, the efficiency of a single junction PSC has reached 25.7% [1], the efficiency of perovskite/perovskite tandem solar-cell PCE is 26.4% [2], and the efficiency of perovskite/silicon tandem solar-cell PCEs is 29.80% [3]. PSCs have become the most popular research objects in the field of solar cells due to their amazing photoelectric properties, such as their great light-absorption coefficient [4], long carrier transmission distance [5], large tolerance factor [6], film super flexibility [7], simple preparation process, and low production cost [8].

The final step in the industrial production of PSCs that combines the advantage of very high PCE, amazing performance, simple fabrication process, and low price of materials is stability. It is known that defects are a key constraint in perovskite devices’ performance and degradation, particularly when the perovskite films are prepared using low-temperature solvent processes. These defects cause the decomposition and the non-radiative recombination of perovskite film, which have a seriously negative impact on PCE and stability [9]. Therefore, various kinds of techniques have been developed to produce efficient PSCs with fewer defects. The next step is to fix this problem and apply this super-solar cell. Improving the preparation process is the most promising way to effectively achieve this. The all-vacuum preparation of perovskite solar cells can effectively avoid the defects caused by solvents, which is the key preparation method to obtain highly stable and large-area devices. Additionally, in the era of rapid scientific and technological progress, vacuum-coating technology has also been updated and iterated. If the existing technology is combined with the preparation of perovskite solar cells, it will be easy to solve the stability problem of perovskite solar cells.

Vacuum deposition has many advantages. First, has low impurity defects, which can prevent the mixing of impure ions and effectively reduce the charge recombination center. Second, the film thickness is easy to control, which means that the thickness of perovskite film can be accurately controlled and the poor repeatability of perovskite film prepared using the solution method can be avoided. Third, the simple preparation process of perovskite films, which only need to evaporate the precursor materials and anneal at their crystallization temperature. Additionally, vacuum processing can prevent waste of raw materials, without any solvent, and roll-to-roll. However, vacuum evaporation needs expensive equipment, which can easily increase the cost of the product. To date, due to technical limitations, PSCs have not yet been commercialized. Excellent PSCs need to be combined with advanced vacuum coating technology to promote the industrial application and achieve its advantages. Although PSCs have obtained great progress at vacuum deposition in the past few years, some theoretical studies also needed to be solved, and the commercial application will be realized in the near future.
